# Epidemiological profile of pregnant women undergoing first-trimester preeclampsia screening at a tertiary reference center in Brazil

**DOI:** 10.1590/1806-9282.20251838

**Published:** 2026-06-19

**Authors:** Vivianne Netto Alves dos Reis, Edward Araujo, Beatriz Bussi Rosolen, Gustavo Yano Callado, Sandra Rejane Silva Herbst, Ingrid Schwach, Carolina Leite Drummond, Giselle Darahem Tedesco

**Affiliations:** 1 Faculdade de Ciências Médicas da Santa Casa de São Paulo, Department of Obstetrics and Gynecology – São Paulo (SP), Brazil.; 2 Universidade Federal de São Paulo, Escola Paulista de Medicina, Department of Obstetrics – São Paulo (SP), Brazil.; 3 Universidade Municipal de São Caetano do Sul, Discipline of Women’s Health – São Caetano do Sul (SP), Brazil.; 4 Hospital Israelita Albert Einstein, Albert Einstein Israelite Faculty of Health Sciences – São Paulo (SP), Brazil.

**Keywords:** Preeclampsia, First trimester, Mean arterial pressure, Risk factors, Maternal health

## Abstract

**OBJECTIVE:**

The aim of this study was to evaluate the epidemiological profile of first-trimester pregnant women attending a tertiary reference center in São Paulo, Brazil, and to assess its association with the development of preeclampsia.

**METHODS:**

This study involved retrospective and prospective cohorts of pregnant women undergoing first-trimester ultrasound for aneuploidy screening between 11 and 13+6 weeks’ gestation from 2020 to 2023. Maternal, obstetric, and clinical characteristics, including mean arterial pressure, were collected via questionnaire and clinical measurements. Statistical analyses included descriptive statistics and stepwise forward logistic regression to identify factors associated with preeclampsia development.

**RESULTS:**

A total of 104 pregnant women were included, with a mean age of 30.9 years. Most were White (48.3%), non-smokers (86.5%), overweight (mean body mass index: 28.5 kg/m2), and had spontaneous pregnancies (100%). Nulliparous and primiparous women accounted for 71.9% of the sample. Low risk for preeclampsia was observed in 84.3% and high risk in 15.7%. History of previous preeclampsia (OR 46.0, 95%CI 3.2–654.2), chronic arterial hypertension (OR 11.2, 95%CI 1.9–64.6), and increased first-trimester mean arterial pressure (OR 1.2, 95%CI 1.1–1.4) were independently associated with higher preeclampsia risk. Other variables, including race, parity, body mass index, and uterine artery Doppler indices, were not significant predictors.

**CONCLUSION:**

Prior history of preeclampsia, chronic hypertension, and elevated first-trimester mean arterial pressure were key predictors of preeclampsia development. These findings support the importance of first-trimester risk stratification using maternal history and mean arterial pressure measurement to guide early preventive interventions, particularly in settings where biochemical markers are not routinely available.

## INTRODUCTION

The prevalence of preeclampsia (PE) is higher in regions with lower socioeconomic development, which account for approximately 99% of cases worldwide. In Brazil, PE affects 1.5–7% of pregnancies, with 0.6% progressing to eclampsia, although these estimates likely vary by region and may be underestimated^1,2^. PE is a multisystem disorder associated with longterm maternal morbidity, including increased risks of stroke, cardiovascular disease, and diabetes, as well as adverse neonatal outcomes primarily related to prematurity, such as perinatal mortality and impaired neurodevelopment^3^.

According to the International Society for the Study of Hypertension in Pregnancy (ISSHP), PE is defined by new-onset hypertension (≥140/90 mmHg) after 20 weeks of gestation in previously normotensive women, accompanied by proteinuria, maternal organ dysfunction, and/or placental involvement^2^. PE may be classified as preterm (<37+0 weeks) or term (≥37+0 weeks), while pathophysiological studies often distinguish early-onset (<34 weeks) from late-onset disease.

First-trimester screening is central to PE prevention, as interventions such as acetylsalicylic acid and calcium supplementation reduce the incidence of preterm PE^4^. The Fetal Medicine Foundation (FMF) model, validated internationally, combines maternal characteristics, medical and obstetric history, biochemical markers, and biophysical parameters, including mean arterial pressure (MAP) and uterine artery Doppler, to identify women at increased risk^1,5^. In Brazil, the Brazilian Federation of Gynecology and Obstetrics Associations (FEBRASGO) guidelines emphasize universal screening based on medical history and MAP, tailored to local resource availability^6^.

Given the substantial maternal and fetal burden of PE and the potential for early prevention, this study aimed to characterize the epidemiological profile of first-trimester pregnant women attending a tertiary reference center in São Paulo, Brazil, and to assess its association with the subsequent development of PE.

## METHODS

This was a descriptive, observational study involving retrospective and prospective cohorts of pregnant women attending the Fetal Medicine Unit, Department of Obstetrics and Gynecology, Santa Casa Hospital, São Paulo, Brazil, between 2020 and 2023. The inclusion criterion was singleton pregnancies undergoing first-trimester ultrasound for aneuploidy screening (gestational age between 11 weeks and 13 weeks+6 days). Exclusion criteria included multiple pregnancies, fetal malformations, Müllerian anomalies, and miscarriages.

PE risk was calculated using the FMF first-trimester algorithm. Women were classified as high risk if the estimated risk for preterm PE (<37 weeks) was ≥1:100, and low risk if <1:100. MAP was measured using a validated automated oscillometric device, following FMF recommendations. After ≥5 min of rest in the seated position, two measurements were obtained from each arm with the appropriate cuff size and arms supported at heart level. The average of the four readings was used to calculate MAP.

Data were collected through a questionnaire administered at the time of the ultrasound examination, including socioeconomic variables (age, race, and smoking status); obstetric variables (gestational age determined by crown–rump length, parity, type of delivery, and prematurity); clinical variables (gestational diabetes mellitus, previous gestational diabetes, chronic arterial hypertension, systemic lupus erythematosus, antiphospholipid syndrome, history of PE in a previous pregnancy, maternal family history of PE, and history of previous small-for-gestational-age fetus); maternal weight, height, and body mass index (BMI); and combined MAP.

Statistical analysis was performed using SPSS version 25.0 (SPSS Inc., Chicago, IL, USA). For descriptive analysis, absolute (n) and relative (%) frequencies were calculated for qualitative variables, and summary measures (mean, standard deviation, median, minimum, and maximum) were calculated for quantitative variables. Chi-square or Fisher’s exact tests were applied to qualitative predictor variables when appropriate, and statistically significant associations were expressed as odds ratios (ORs) with 95%CIs. For quantitative predictors, the Student’s t-test or Mann-Whitney test was used according to data distribution. A significance level of 5% (p<0.05) was adopted for all tests.

For multivariate analysis, predictors with p<0.20 in univariate analysis were preselected, followed by stepwise forward logistic regression to identify clinically significant variables associated with the development of PE. Among these, chronic arterial hypertension, combined MAP, and a history of PE in a previous pregnancy were identified.

Normality was assessed using the Shapiro-Wilk test. Parametric or nonparametric tests were applied accordingly. MAP was analyzed as a continuous variable. Missing data were minimal (<5%) and handled by complete-case analysis.

## RESULTS

In our study, 104 pregnant women who underwent a first-trimester scan for aneuploidy screening with concomitant risk calculation for PE were evaluated. The mean maternal age was 30.9 years, with most participants being White (48; 41.6%) and non-smokers (91; 87.5%). The mean BMI was 28.5 kg/m^2^ (range: 16.6–48.6 kg/m^2^), indicating that most women were overweight, and all pregnancies were spontaneous (104; 100%). The most frequent gestational age at examination was 12–13 weeks (72; 69.7%). Regarding parity, nulliparous and primiparous women together accounted almost all sample (37 nulliparas; 66 primiparas; total 103; 99.0%). Most women had no previous history of PE (98; 94.2%), chronic arterial hypertension (85; 81.7%), gestational diabetes mellitus (98; 94.2%), preexisting diabetes mellitus (96; 92.3%), or maternal history of PE (95; 91.3%) (Table 1).

**Table 1:** Maternal and gestational characteristics.

Maternal characteristics	n (%)
Maternal age (years)	
<35 years	70 (67.3)
≥35 years	34 (32.7)
Body mass index (kg/m^2^)	
Normal (18.5–25)	22 (21.15)
Overweight (≥25)	78 (75)
Underweight (<18.5)	4 (3.85)
Race	
White	48 (46.1)
Black	14 (13.5)
Mixed	42 (40.4)
Smoking	
Yes	13 (12.5)
No	91 (87.5)
Parity	
Nulliparous	37 (35.6)
Primiparous	66 (63.5)
Multiparous (≥2)	1 (1.0)
Chronic arterial hypertension	
Yes	19 (18.3)
No	85 (81.7)
Previous diabetes mellitus	
Yes	8 (7.7)
No	96 (92.3)
Gestational diabetes mellitus	
Yes	6 (5.8)
No	98 (94.2)
Previous preeclampsia	
Yes	6 (5.8)
No	98 (94.2)
Maternal history of preeclampsia	
Yes	9 (8.7)
No	95 (91.3)
Conception	
Spontaneous	104 (100)
Assisted	0 (0)

When these parameters were evaluated, we found a low-risk rate for PE of 84.3% and a high-risk rate of 15.7%, although without statistical significance. Among the factors identified as being associated with a higher risk of PE development, a history of previous PE showed the strongest association (p=0.005), with an OR of 46.0 and a 95%CI 3.2–654.2, suggesting that pregnant women with this antecedent have an approximately 46-fold higher risk of developing PE in the current pregnancy. The presence of chronic arterial hypertension also demonstrated a strong association (p=0.007), with an OR 11.2 and a 95%CI 1.9–64.6. Additionally, an increase in combined MAP was associated with a progressive risk of PE (p=0.006), with an OR 1.2 and a 95%CI 1.1–1.4, reinforcing the importance of MAP measurement as an early screening tool (Table 2). Other analyzed variables, such as race, parity, BMI, weight, height, and mean uterine artery pulsatility index (PI mean), did not show statistical significance (Table 3).

**Table 2:** Maternal characteristics and risk for early-onset preeclampsia.

	p-value	OR	95%CI
Chronic arterial hypertension	0.007	11.2	1.9–64.6
Combined mean arterial pressure	0.006	1.2	1.1–1.4
Previous preeclampsia	0.005	46.0	3.2–654.2

CI: confidence interval; OR: odds ratio.

**Table 3:** Quantitative variables and risk for early-onset preeclampsia.

Quantitative characteristics	Low risk mean (SD)	High risk mean (SD)	p-value
Height (m)	1.63 (0.07)	1.61 (0.06)	0.44
Weight (kg)	75 (18.0)	79 (17)	0.385
Mean arterial pressure (mmHg)	83.7 (75)	100 (12.0)	<0.001
Mean uterine pulsatility index of uterine arteries Doppler	21 (27)	19.2 (29.0)	0.673

SD: standard deviation.

## DISCUSSION

In this first-trimester cohort of 104 pregnant women, the prevalence of high risk for PE was low (15.7%); however, three maternal factors were independently associated with disease development: previous PE, chronic arterial hypertension, and elevated MAP. A history of PE conferred the highest risk, with an approximately 46-fold increased likelihood of recurrence, followed by chronic arterial hypertension (11-fold increase). Incremental increases in MAP were also associated with progressively higher risk. In contrast, age, BMI, race, and parity were not significantly associated. These findings highlight the predictive value of clinical history and hemodynamic assessment using simple, reproducible parameters in early prenatal care.

Our findings that a prior history of PE is the strongest predictor of recurrence are consistent with previous cohort studies, which report recurrence rates ranging from 14 to 21%, with higher rates (up to 31%) for early-onset or severe cases^7,8,9,10^. The magnitude of association observed in our cohort (OR 46.0) aligns with prior reports indicating substantially increased adjusted relative risks or incidence rate ratios (aRRs/IRRs: 12.8– 14.5) for recurrent PE, particularly for preterm disease or when combined with interpregnancy hypertension^7,9,10^. Additionally, comorbidities such as chronic hypertension, obesity, and diabetes further elevate the risk, supporting the multifactorial nature of recurrence. These results reinforce the clinical relevance of obtaining a detailed obstetric history during the first trimester and are in line with validated predictive models, including the FMF algorithm, which integrates maternal history, MAP, uterine artery Doppler, and placental growth factor (PIGF) to identify high-risk women early^7,10^. Importantly, although recurrence risk is high, most women with prior PE do not experience recurrence, and outcomes in subsequent pregnancies are generally favorable with individualized monitoring and prophylactic interventions, such as low-dose aspirin initiated before 16 weeks’ gestation^10^.

Mean uterine artery PI was not significantly associated with PE risk. The wide variability observed suggests limited standalone predictive value in this cohort, consistent with evidence that uterine artery Doppler performs best when combined with clinical and biochemical markers.

The association of elevated first-trimester MAP with increased PE risk observed in our study is also supported by international evidence. MAP alone has demonstrated moderate predictive performance, with area under the curve (AUC) values ranging from 0.82 to 0.87 for overall and term PE, and up to 0.91 for later gestational ages in some populations^11,12^. However, predictive accuracy improves markedly when MAP is incorporated into multiparametric models alongside maternal characteristics, uterine artery Doppler, and angiogenic biomarkers such as PlGF, as in the FMF triple test, achieving detection rates of 75–90% for early-onset and preterm PE^13,14,15^. Our finding that MAP is independently associated with progressively increased risk (OR 1.2) underscore its utility as a simple, low-cost, and accessible early screening tool, particularly in settings where biochemical markers may not be routinely available^11,13,14,15^. These results corroborate previous studies demonstrating that first-trimester MAP measurement is a robust component of risk stratification and can guide timely prophylactic interventions.

Advanced modeling of uterine artery PI using fractional polynomials has improved risk estimation but remains insufficient alone. Increased PE risk has also been associated with spontaneous preterm and term birth in unaffected pregnancies, whereas iatrogenic preterm birth remains strongly linked to placental dysfunction in established disease^16,17^.

This study has several limitations that should be acknowledged. First, the relatively small sample size and single-center design may limit the generalizability of the findings to other populations and healthcare settings. Second, as data collection was based partly on self-reported information, potential recall or reporting bias cannot be excluded. Third, biochemical markers such as PlGF or pregnancy-associated plasma protein-A (PAPP-A) were not incorporated into the analysis, which may have restricted the comprehensive assessment of the FMF screening model. Stepwise regression may introduce selection bias; thus, results should be interpreted cautiously. The absence of biochemical markers (PlGF, PAPP-A) likely reduced screening performance, as their inclusion improves detection rates in the FMF model. Nonetheless, this reflects real-world Brazilian settings where such markers are often unavailable. Despite these limitations, the study provides relevant epidemiological insights into first-trimester PE screening in a Brazilian tertiary care setting, contributing to the optimization of local prenatal care strategies.

## CONCLUSION

Our findings support the use of maternal history and standardized MAP measurement as feasible first-trimester screening tools, particularly in Brazilian settings with limited access to biochemical markers. Although limited by its sample size, the findings highlight the importance of incorporating standardized first-trimester screening protocols into routine prenatal care to improve maternal and perinatal outcomes. Further multicenter studies with larger cohorts are needed to validate these results and to refine risk prediction strategies in the Brazilian population.

## Data Availability

The datasets generated and/or analyzed during the current study are available from the corresponding author upon reasonable request.
